# Our New York Letter

**Published:** 1891-02

**Authors:** 


### Correspondence.

### OUR NEW YORK LETTER.

NEW YORK, JAN. 18, 1891.

The enthusiasm which greeted the first announcement of Koch's treatment of tuberculosis has not abated in the least in this city. Many of the hospital physicians who made hurried visits to Berlin, have returned ; demonstrations of the method have been given at various hospitals here, and numerous papers on the subject have been read at the Academy of Medicine meetings. All who seem to know anything of the subject are in one accord with regard to the virulent nature of this fluid and the necessity of limiting its use to certain forms of tuberculosis. It is probable now that its therapeutic influence will be restricted to tuberculous affections of the joints and lupus, while it is very questionable whether it will prove at all efficacious even in the first stage of pulmonary phthisis. The results obtained by the injection in this city have been on the whole confirmatory of Dr. Koch's statement with regard to the therapeutic limitations of the fluid.

It is, however, too soon to judge of the exact value of this treatment, and it is to be hoped that time will serve to demonstrate the efficacy of a remedy for which so much has been claimed. The treatment is at present undergoing evolution, and it is rather too soon to speak of its efficacy or its failure in tubercular affections. Unquestionably the most striking of the local effects of the injections have been witnessed in cases of lupus treated in this city. In not a single case of this affection has the characteristic reaction been wanting ; great inflammatory œdema, followed by exudation and destruction of the lupoid nodules has invariably occurred, and reparative processes have been set up with surprising rapidity.

Many cases of tuberculous glands and joint affections have also been treated in the hospitals here within the past week by this new method, but with less uniform results than in the case of lupus. Tubercular laryngitis has been subjected to this procedure, and for the most part with much the same characteristic symptoms as in the case of lupus. The cases of pulmonary phthisis treated here have been, to say the least, unsatisfactory; whether this be due to the advanced stage of the disease in the cases experimented upon, or to the difficulty of determining by physical signs the degree of local reaction that has taken place, it is impossible to say.

Prof. Robert F. Weir gave last week at the New York Hospital a very interesting demonstration on the subject of finger nail bacteria. He first washed his hands thoroughly with soap and water, and then immersed them for five minutes in a sublimate solution of 1-1000. The scrapings of the nails were then examined by the microscopist of the hospital, and he reported that he found therein large colonies of bacteria. The finger nails were subsequently immersed for one minute in alcohol solution and the scrapings again subjected to the microscopical examination, this time with the result of no bacteria being present. In other words, the demonstration proved this one essential fact, that sublimate solution does not act as disinfectant unless supplemented by alcohol. It is therefore an important thing for surgeons to remember, that by an immersion of their hands for five minutes in a sublimate solution of 1-1000 and one minute in alcohol, they can secure a perfect antisepsis. Dr. Weir stated that the London surgeons were in the habit of using tincture of iodine in the proportion of one ounce to a pint as a final wash instead of alcohol, and that they regard this as a powerful disinfectant. The lecturer in this connection referred to carbolic acid as being the most powerful and certain of germicides in the proportion of 1-20, and that he had secured the best results from its use. It has the disadvantage, however, that you can never be sure of its strength, and that it is besides a very poisonous substance.

At the last meeting of the Section of Surgery of the New York Academy of Medicine, Dr. Willy Meyer showed a patient from whom he had removed the entire larynx for epithelioma of that organ. The patient was a male, aged 65, who had been admitted to the German Hospital on September 27th, suffering from hoarseness and dyspnoea. His history showed that about six months previous to his admission to the hospital, the hoarseness commenced, and that dyspnoea had been present for about ten days. On the eleventh day following his admission to the hospital the difficulty in breathing had become so marked that tracheotomy had to be performed at once, under chloroform. Before making the incision necessary to perform the operation, a hard mass was felt in the jugular space, and on cutting down to it, it was found to be an osseous growth, apparently adherent to the anterior surface of the thyroid cartilage. This was removed by means of the bone forceps after a good deal of trouble. As a result the dyspnoea was at once relieved, and the patient experienced much relief after the insertion of the tube into the trachea. During the following day he had a bronchitis, with high fever, but soon recovered. A careful examination of the larynx revealed an irregular mass on the right side, involving the vocal cords.

Tuberculosis was excluded in this case, and the patient was put on anti syphilitic treatment for three weeks, but without any material benefit. The entire larynx was finally removed, the epiglottis being left behind, as it seemed to be in a normal condition. The wound was packed with iodoform gauze after the operation. On the fifth day a slight rise of temperature occurred, which disappeared on the following day. About forty days after the operation an artificial larynx was introduced, and the patient bears it well. The patient made several attempts to talk at the meeting, but the speech was unintelligible. The doctor thought that in time, however, he might improve in this respect.

Dr. Townsend at the same meeting, exhibited a colored girl, seventeen years of age, with complete dislocation of the left patella. It was believed to be congenital, and he stated there were only fifteen or twenty such examples on record. The patient had also some of knock-knee. The only treatment in this case was a well fitting bandage, and a knee cap, which maintained the patella in position. As a result of this treatment, the patella could now be dislocated in only one direction, (outward). The patient experienced no difficulty from this trouble, except that occasionally in walking, the patella would spring from one side to another, and the leg would become stiff as a result.

Dr. F. Curtis presented at a recent clinic, a carpenter, with a refracture of the radius. About a year ago he sustained a

Collis fracture at the wrist. He was treated for this and a good result secured. Five weeks after the injury the splint was removed. Two months later he was climbing a ladder, and missing a few steps, he slipped and caught on to the ladder with his left hand to save himself from falling. As a result, he reproduced the former fracture. Dr. Curtis saw him two days after the injury, and found free mobility and decided crepitus. Refracture of the radius, Dr. Curtis stated was so rare that he considered it of very great interest. In about forty cases of secondary fracture of the bones, the great majority occurred in the femur and tibia, only three occurring in the forearm. That traction should bring about a refracture he considered a very interesting fact. P. J. R.

---

CASE OF RECTAL OBSTRUCTION IN A CHILD.—T. Sympson (British Medical Journal, October 4, 1890). "A boy, æt. ten years, while spending a week with some relations in the country, ate a large quantity of wheat. The day after his return home he was noticed to have lost his appetite, and to be listless. In the evening he suffered greatly from abdominal pain, frequent and urgent desire to evacuate his bowels, and severe tenesmus. These symptoms increased in intensity. On the third day I was called to see him. On examination through the abdominal wall, the sigmoid flexure was felt to be greatly distended, and a few grains of wheat had been found in the bed. On examination, liquid was seen oozing from the anus, and the rectum was enormously distended. Under chloroform, a quart pot of wheat was removed, with complete relief to all the symptoms.—*Brooklyn Medical Journal.*

---

THE TREATMENT OF NEURALGIA SPERMATICA.—Dr. T. Benda (Berliner klin. Wochenschrift, No. 38, 1890) writes of a patient affected with a severe neuralgia of the spermatic cord on whom the operation of castration had been employed in vain. After a slight mitigation of the pain from the use of electricity, a very favorable result was obtained from an apparatus similar to a truss, so constructed as to exercise permanent pressure over the vas deferens and the inguinal region.—(*Jour. Cutaneous and Genito-Urinary Diseases.*)

